# OSgc: A Web Portal to Assess the Performance of Prognostic Biomarkers in Gastric Cancer

**DOI:** 10.3389/fonc.2022.856988

**Published:** 2022-03-11

**Authors:** Longxiang Xie, Qiang Wang, Zhongyi Yan, Yali Han, Xiaoyu Ma, Huimin Li, Lu Zhang, Xianzhe Li, Xiangqian Guo

**Affiliations:** ^1^ Department of Preventive Medicine, Institute of Biomedical Informatics, Cell Signal Transduction Laboratory, Bioinformatics Center, Henan Provincial Engineering Center for Tumor Molecular Medicine, School of Software, School of Basic Medical Sciences, Henan University, Kaifeng, China; ^2^ Department of Thoracic Surgery, The Affiliated Nanshi Hospital of Henan University, Nanyang, China

**Keywords:** gastric cancer, gene expression, GEO, OSgc, prognostic biomarker, survival analysis, TCGA

## Abstract

Evaluating the prognostic value of genes of interest in different populations of gastric cancer (GC) is difficult and time-consuming for basic and translational researchers even though many datasets are available in public dataset depositories. In the current study, we developed a robust web-based portal called OSgc (Online consensus Survival analysis of gastric cancer) that enables easy and swift verification of known and novel biomarker candidates in GC. OSgc is composed of gene expression profiling data and clinical follow-up information of 1,824 clinical GC cases, which are collected from 7 public independent datasets derived from Gene Expression Omnibus (GEO) and The Cancer Genome Atlas (TCGA). By OSgc, users input the official gene symbol and will promptly retrieve the Kaplan–Meier survival plot with hazard ratio (HR) and log rank *p* value on the output webpage, by which users could assess the prognostic value of interesting genes for GC patients. Five survival end points containing overall survival, progression-free survival, progression-free interval, relapse-free survival, and disease-free survival could be measured in OSgc. OSgc can greatly help cancer biologists and clinicians to explore the effect of gene expression on patient survival. OSgc is freely available without restrictions at http://bioinfo.henu.edu.cn/GC/GCList.jsp.

## Introduction

Gastric cancer (GC) is the fourth leading factor of cancer mortality in the world. In 2020, GC occurred in 1,089,103 people and resulted in 768,793 deaths. Although there are many advances in treatment of GC, patients have poor prognosis and the 5-year survival rate is just 5%–20%. Prognostic biomarkers can assist clinicians in assessing the risk of clinical outcomes including cancer recurrence or disease progression in the future (1, 2). Molecular characteristics such as gene expression and somatic mutations have been reported to represent the primary source of prognostic biomarker (3, 4). A recent study showed that high *SETD2* (SET domain-containing protein 2, also known as *HYPB*) expression was correlated with better prognosis for GC patients, and its overexpression in GC cell lines significantly inhibits cell proliferation, migration, and invasion (5). Moreover, lower *MTBP* (MDM2-binding protein) expression and *HOXA5* (homeobox A5) expression were significantly associated with longer overall survival time in GC (6). However, these present retrospective cohort studies were limited to relatively small case series and further validation is required when these findings are going to be translated.

The expression profiling of gastric cancer has been performed using high-throughput technology such as microarrays and RNA sequencing. These data have been used to measure the association of mRNAs to clinical outcomes in GC patients, while a key step for biomarker development is the biomarker validation in multiple independent cohorts. Even though massive public datasets are available, multistep specialized analyses such as exploring repositories and acquiring and processing data make it difficult for most researchers. Previous studies have reported several good online prognosis tools including PROGgene (7), PRECOG (8), OncoLnc (9), GEPIA (10), KM plotter (11, 12), and ITTACA (13), which are available to explore expression changes of individual genes and their association with GC patients’ survival. However, these above tools are restricted regarding low number of clinical cases, lacking most updated data, or login/registering, or limited survival terms.

Hence, to aid and facilitate the evaluation and verification of prognostic biomarkers in independent cohorts, we developed OSgc, a free and easy-to-use web portal to perform the survival analysis in GC. OSgc is composed of 7 public datasets with available follow-up data for 1,824 GC cases from TCGA and GEO databases, and by which survival analysis can be completed in minutes.

## Material and Methods

### Data Collection

Data were collected by searching for keywords related to gastric cancer, clinical outcome, mRNA profiles, and ≥20 samples. The searches were performed in GEO (http://www.ncbi.nlm.nih.gov/geo) and TCGA (https://tcga-data.nci.nih.gov/tcga). TCGA data are level 3 RNA-Seq data.

### Web Implementation

**Figure 1:**
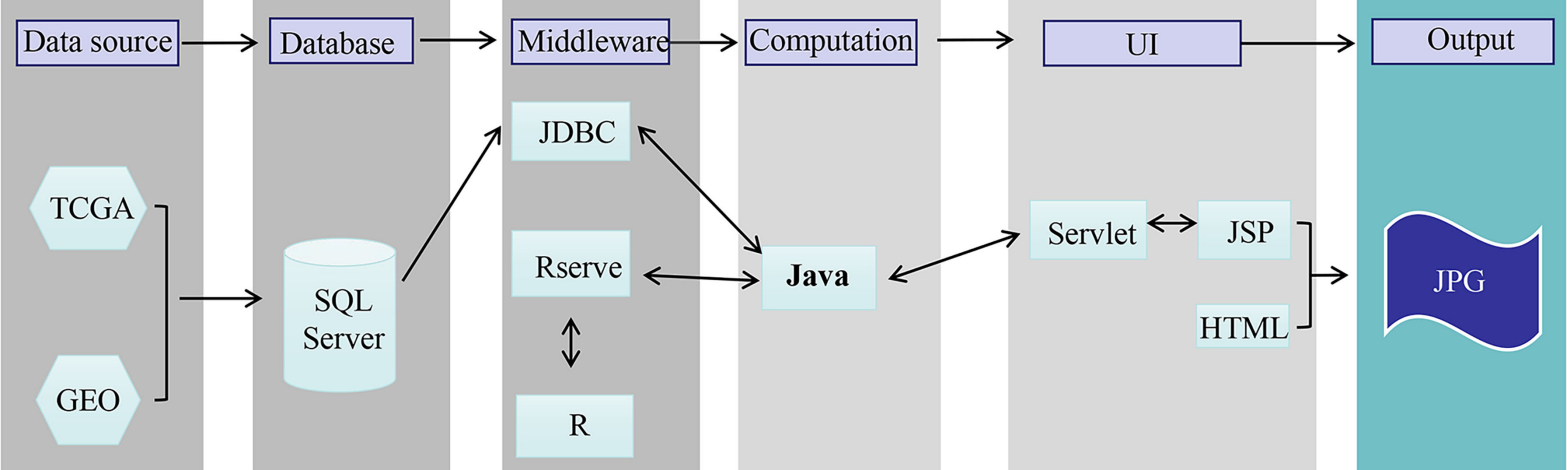
The flowchart of OSgc establishment. GEO, Gene Expression Omnibus; TCGA, The Cancer Genome Atlas.

The OSgc web portal was set up as we previously developed with minor modification (14, 15). In brief, OSgc contains two main components: storage and data analysis (**Figure 1**). A Java implementation was used to realize OSgc. OSgc used the SQL Server database to provide the storage and management of the gene expression profiles and clinical data for GC and used the Browser/Server architecture network management system to manage the database. R packages including “survminer” and “ggplot2” were used to plot the Kaplan–Meier curves.

### Searching Previously Reported Prognostic Biomarkers

A PubMed search was performed to find gastric cancer’s prognostic biomarkers using keywords “gastric cancer,” “stomach cancer,” “adenocarcinoma,” “GC,” “survival,” and “gene expression.” Only studies published in English were encompassed. Eligibility criteria also contained the investigation of the biomarker in >30 patients while biomarkers described only in experimental models or meta-analysis or bioinformatics were excluded.

### Statistical Analysis

The relationship between clinic-pathological factors and clinical survival outcomes was analyzed by GraphPad Prism 8. R package “survival” was used to perform Cox regression analysis to calculate hazard ratios (HR) and 95% confidence intervals (95% CIs). HR with their corresponding 95% CIs was assessed to explore the prognostic significance of gene of interest in gastric cancer. If a HR is >1 and the 95% CI did not cross 1, the typed gene will show a worse prognosis in the high gene expression group. If a HR is <1 and the 95% CI did not cross 1, it suggests a better prognosis of typed gene in the high gene expression group. Prognostic value was assessed by KM plot analysis and log-rank test. *p* value < 0.05 is regarded as statistically significant.

## Results

### Clinical Information of GC Datasets Used in OSgc

To our knowledge, OSgc provides the largest compilation of expression profiling datasets related to clinical outcomes, comprising 7 datasets and 1,824 clinical GC cases. The median age of these patients is 67 years, and the ratio of male to female is 2.2:1. 1,392 patients have OS, 776 patients have DFS, 420 patients have PFS, and 337 patients have RFS. A summary of above GC cohorts is shown in **Table 1**.

**Table 1:** Clinical characteristics of individual dataset in OSgc.

ID	GPL	Sample size	Gender Female/male	Number of deaths	Grade	Stage (I/II/III/IV)	Median age (years)	Survival terms	Reference
GSE22377	GPL570	43	28/15	30	–	2/12/19/2	64	OS, DFS	(16)
GSE26253	GPL8432	432	–	–	–	68/167/130/67		RFS	(17)
GSE29272	GPL96	126	27/99	31	1/44/81	5/4/108/8	59	OS	(18)
GSE57303	GPL570	70	14/56	7	–	0/4/34/32	68	OS	(19)
GSE62254	GPL570	300	101/199	152	–	30/96/95/77	64	OS, DFS	(20)
GSE84437	GPL6947	433	137/296	209	–	11/38/92/292	62	OS	(21)
TCGA-GC	RNAseq	420	134/286	169	10/150/251^a^	55/128/202/40	67	OS, RFS, PFI, PFS	(20)
Total		1824	441/951	598	11/194/332	171/593/677/519			

a 9 samples were GX that grade cannot be defined.

**Figure 2:**
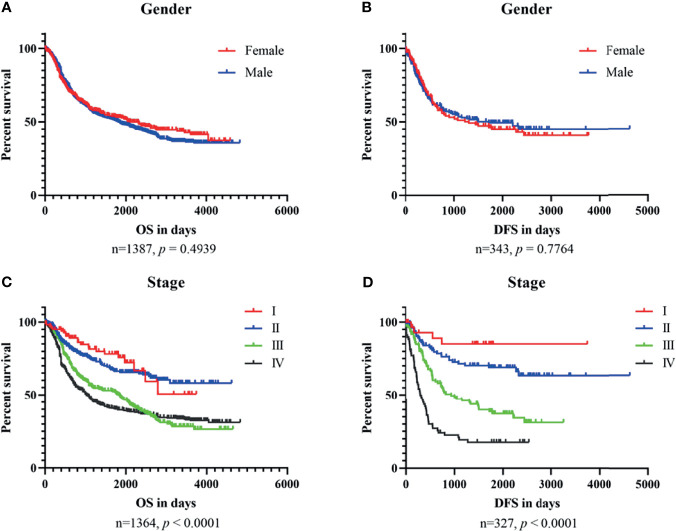
The association of GC patient survival with gender **(A, B)** and stage **(C, D)** in OSgc.

To explore the relationship between clinical characteristics and outcomes, we performed Kaplan–Meier plots for GC patients stratified by TNM stage and gender in datasets used for OSgc (**Figure 2**). In these patients, TNM stage was significantly related with OS (*p* < 0.0001) and DFS (*p* < 0.0001), as we knew (22). Nevertheless, gender showed no significant association with OS (*p* = 0.4939) and DFS (*p* = 0.7764).

### Web Interface

**Figure 3:**
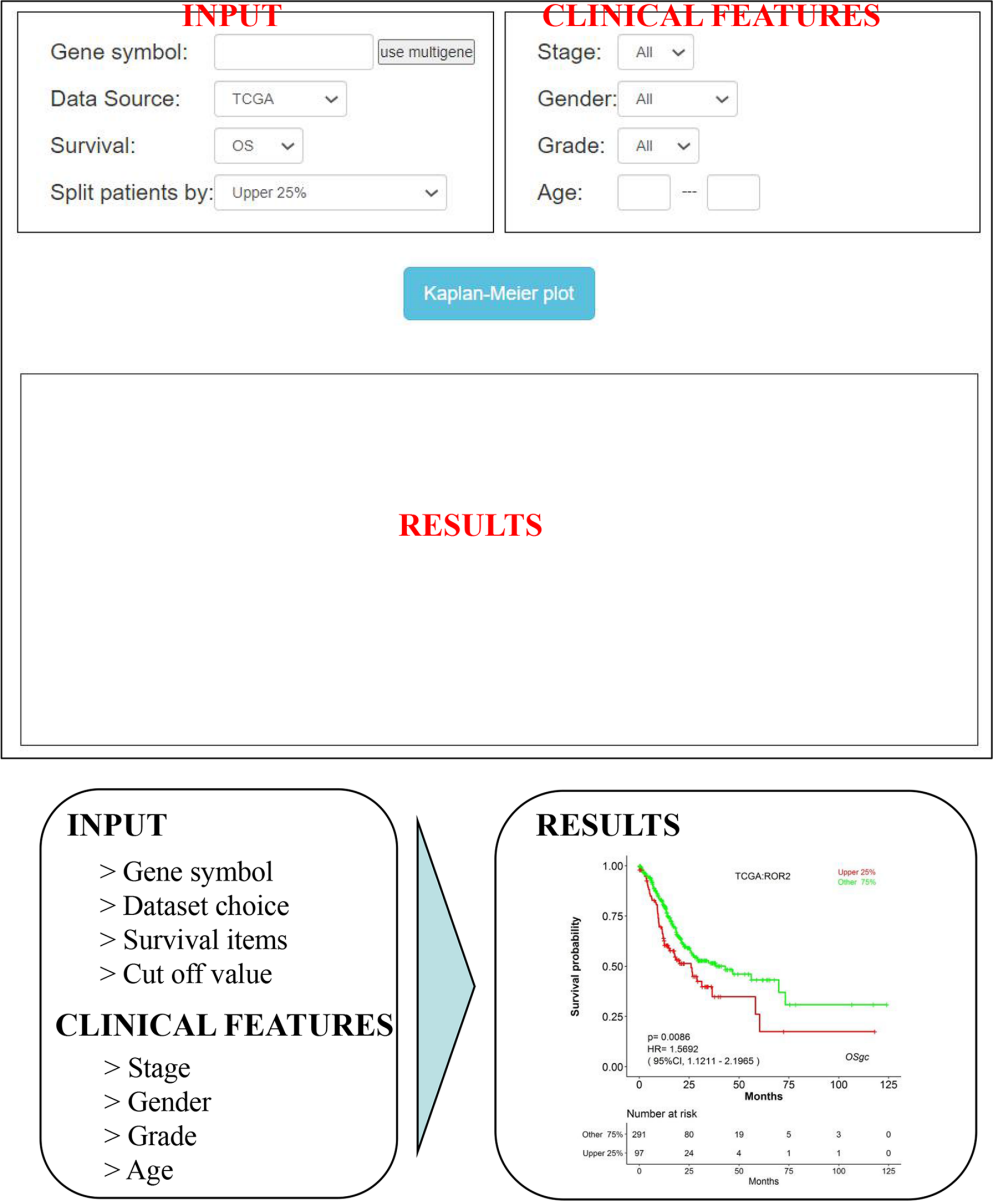
An overview of OSgc application. OSgc application mainly contains three sections including input, clinical features, and results.

As shown in **Figure 3**, OSgc could be easily used by typing only the gene symbol and selecting the individual/combined cohort, stage, gender, grade, and age on the input webpage. After then, “Kaplan–Meier plot” is clicked, and the survival outcome plots will be shown up on the output webpage in less than a minute (might vary if advanced plots are selected). Furthermore, users can easily obtain KM plots for multiple genes (with maximum 5 genes).

### Validation and Application

To assess the capabilities of OSgc in the evaluation of prognostic biomarkers, we collected 20 previously published GC prognostic biomarkers through PubMed search (shown in **Table 2**). These published prognostic biomarkers include 16 unfavorable and 4 favorable prognostic biomarkers which have been verified by tissue-based immunohistochemistry (IHC), Western blot, and RT-PCR in literatures. To test these published prognostic biomarkers in OSgc, OS or DFS was selected as survival term in the combined datasets in OSgc. Combined datasets mean that each cohort was divided separately into strata by selecting the proper cutoff value, which are then put together for survival analysis. The hazard ratio and *p* value in original studies and OSgc are given in **Table 2**. The test results showed that the prognostic values of 17 genes line up with previous reports, while 3 genes did not reach significance in OSgc.

**Table 2:** Test the performance of previously reported prognostic biomarker candidates using OSgc.

Gene symbol^a^	Literature data					OSgc data			
	N	Survival	Prognostic value	Method	Reference	Dataset	HR (95% CI)	*p* value	Validation results
HOXA5	30	OS	Unfavorable	qRT-PCR and Western blot	(23)	Combined	1.3724 (1.114–1.5818)	0.0015	√
CAP2	436	OS	Unfavorable	RT-PCR	(24)	Combined	1.5207 (1.2645–1.7858)	<0.0001	√
LAMA4	388	OS	Unfavorable	qRT-PCR	(25)	Combined	1.4904 (1.2305–1.8053)	<0.0001	√
MTBP	352	OS	Unfavorable	qRT-PCR and Western blot	(6)	GSE22377	2.7219 (1.2506–5.9242)	0.0116	√
RAI14	68	OS	Unfavorable	IHC	(26)	Combined	1.6117 (1.3304–1.9524)	<0.0001	√
SETD2	153	OS	Favorable	qRT-PCR and IHC	(5)	Combined	0.6946 (1.3304–1.0078)	0.0603	×
NDRG4	286	OS	Unfavorable	IHC	(27)	Combined	1.4149 (1.1904–1.6818)	1e-04	√
SPARC	227	OS	Unfavorable	qRT-PCR	(28)	Combined	1.4217 (1.1612–1.7407)	7e-04	√
HOXB9	190	OS	Favorable	IHC	(29)	Combined	0.8461 (0.7008–1.0215)	0.0821	×
DDC	39	OS	Favorable	qRT-PCR and IHC	(30)	GSE22377	0.2805 (0.097–0.8108)	0.0189	√
ERCC1	106	OS	Unfavorable	qRT-PCR	(31)	GSE62254	1.6054 (1.1377–2.2662)	0.007	√
STAT3	50	OS	Unfavorable	RT-PCR, Western blot and IHC	(32)	TCGA GSE22377	1.4306 (1.0138–2.0188) 3.0762 (1.4271–6.6306)	0.0416 0.0041	√
IGFBP7	247	OS	Unfavorable	qRT-PCR and IHC	(33)	Combined	1.4577 (1.2253–1.7342)	<0.0001	√
TIMP3	17	OS	Unfavorable	qRT-PCR and IHC	(34)	Combined	1.4779 (1.2283–1.7783)	<0.0001	√
KLK6	66	OS	Unfavorable	qRT-PCR and IHC	(35)	GSE62254	1.4878 (1.0523–2.1037)	0.0246	√
NNMT	641	OS	Unfavorable	qRT-PCR	(36)	Combined	1.3734 (1.1529–1.6362)	4e-04	√
ATAD2	166	OS	Unfavorable	qRT-PCR	(37)	GSE22377	3.4084 (1.5908–7.3027)	0.0016	√
CXCR3	96	OS	Favorable	RT-PCR and qRT-PCR	(38)	Combined	0.7203 (0.5923–0.8759)	0.0010	√
SMYD3	166	OS	Unfavorable	IHC	(39)	Combined	1.1857 (0.9798–1.435)	0.0801	×
S100A4	434	OS	Unfavorable	qRT-PCR	(40)	Combined	1.2081 (1.0103–1.4447)	0.0382	√

a HOXA5, homeobox A5; CAP2, cyclase-associated protein 2; LAMA4, laminin a4; MTBP, MDM2 binding protein; RAI14, retinoic acid induced 14; SETD2, SET domain containing; NDRG4, N-Myc downstream-regulated gene 4; SPARC: secreted protein acidic and rich in cysteine; HOXB9: homeodomain-containing transcription factor; DDC: Dopa decarboxylase; ERCC1, excision repair cross complementation group 1; STAT3, signal transducers and activators of transcription; IGFBP7, insulin-like growth factor binding protein 7; KLK6, kallikrein 6; NNMT, nicotinamide N-methyltransferase; ATAD2, ATPase family, AAA domain containing 2; CXCR3, chemokine receptor; SMYD3, MYND domain-containing protein 3; S100A4: S100 calcium binding protein A4.

### Comprehensive Analysis of the Prognosis Significance of E2Fs by OSgc

**Figure 4:**
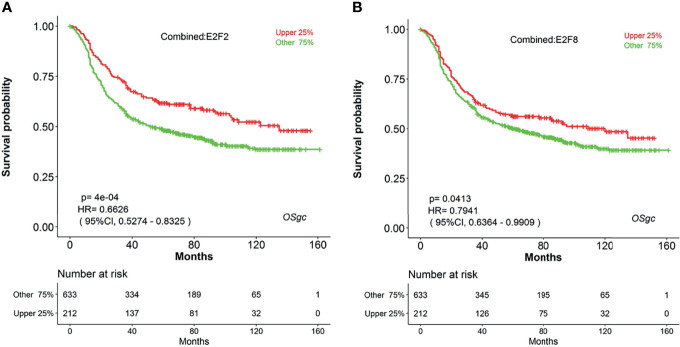
The prognostic value of E2F2 and E2F8 in GC patients (OS in OSgc). **(A)** KM survival plot for E2F2 suggests that its high expression (red) indicates favorable prognosis (GC patients were separated by the quarter of gene expression level); **(B)** KM survival plot for E2F8 suggests that its high expression (red) indicates favorable prognosis (GC patients were separated by the quarter of gene expression level); E2F2, E2F transcription factor 2; E2F8, E2F transcription factor 8; GC, gastric cancer; OS, overall survival.

Increasing evidence has indicated that E2Fs, a family of critical transcription factors that regulate cell cycle progression and other cellular processes, are aberrantly expressed and involved in the tumor progression in various malignant tumors (41, 42). Recently, four research groups had systematically studied the expression patterns and prognostic values of eight E2Fs in patients with breast cancer (BC) (43), ovarian cancer (OC) (44), hepatocellular carcinoma (HCC) (45), and lung carcinoma (46) by investigating a series of databases. For example, Huang et al. had shown that high expression of individual *E2Fs* was related with poor prognosis in HCC patients (45). However, the expression and prognostic significance of each E2F in gastric cancer have not yet been elucidated. Herein, we can easily explore the prognostic significance for all *E2F* members in GC by using web tool OSgc. The results showed that the higher transcriptional levels of both *E2F2* and *E2F8* were associated with better overall survival in gastric cancer patients (**Figure 4**). However, other E2Fs members were not significantly correlated with OS of GC patients (data not shown).

## Discussion

Prognostic biomarkers are an important supplement to traditional clinical and histopathological features, for example, tumor size and lymph node metastasis, which cannot completely predict the prognosis of patients with cancer. The development of biomarkers by genomic, transcriptomic, and proteomic methods holds the promise of “individualized medicine,” bringing a new ground-breaking point to disease diagnosis, classification, and prognosis. The gene expression profiling datasets in TCGA and GEO are of great value in deepening our understanding of the underlying molecular mechanisms involved in GC, as well as in the identification of novel diagnostic and prognostic biomarkers (47, 48). For the maximum utilization of GEO and TCGA data resources, it is very necessary to provide a web-based portal that allows clinicians and cancer biologists (regardless of having bioinformatics background or not) to easily access, analyze, and visualize the data. To identify genes related with patient survival is one possible way to prioritize genes with oncogenic or tumor-suppressor properties for further study. The user-friendly web portal of OSgc promotes the gene identification for survival associations in GC. Compared with published survival web tools such as OncoLnc (9), GEPIA (10), and KM plotter (11, 12), GEPIA and KM plotters are good in performing an extensive survey of prognosis in general cancer types; however, it has limited cases of GC (GEPIA contains 375 patients from TCGA; KM plotter includes 1,440 cases from TCGA and GEO). OncoLnc only containing TCGA data was developed to assess the prognostic significance of non-coding genes. More importantly, OSgc has integrated seven GC cohorts and incorporated the clinical covariates to provide more informative survival plots to the researchers.

We have demonstrated here the usefulness of OSgc using 20 previously published prognostic biomarkers as examples for rapid survival analysis. 85% (17 of 20) of these reported prognostic biomarkers were confirmed to be prognostically significant in OSgc. The three genes without statistical significance in OSgc could be due to several factors including different detecting methods (the analysis method for the *HOXBP* and *SMYD3* prognosis study was IHC) and starting material (the material for *SETD2* prognosis study was paraffin-embedded FFPE tissues). Moreover, we used OSgc to quickly systematically analyze the potential prognostic values of *E2F* family members and found that both *E2F2* and *E2F8* are favorable prognostic biomarkers for GC patients. Previous studies indicated that E2F2 exhibited as a tumor suppressor in epithelial tissues (49) or Myc-induced T cell lymphomagenesis (50), and overexpression of E2F2 inhibited the progression of these tumors. E2F8 is also a critical tumor suppressor for postnatal liver development (51). Compared with other web tools, OSgc is a free tool with the largest number of gastric cancers to perform the survival analysis based on gene expression. In the future, we will check whether the new standard datasets (including gene expression profile and clinical survival information) from different databases (such as TCGA, GEO and ArrayExpress) come out every 3 months, then we will incorporate these new gene expression profiles as well as additional utilities which are suggested by the users.

All in all, OSgc is a free and easy-to-use web portal to assess the performance of potential prognostic biomarkers for gastric cancer.

## Data Availability Statement

The original contributions presented in the study are included in the article/supplementary material. Further inquiries can be directed to the corresponding authors.

## Author Contributions

XG, XL: study concept and design. LX, QW, ZY: acquisition of data. QW, LX, YH, HL, LZ: analysis and interpretation of data. LX, QW, ZY, XG, XL: draft of the manuscript. XG, LX, QW, ZY, XL: critical revision of the manuscript for intellectual content. All authors contributed to the article and approved the submitted version.

## Funding

This work was supported by the National Natural Science Foundation of China (No. U2004136), supporting program for Central Plain Young Top Talents (ZYQR201912176), Kaifeng Science and technology development Plan (2103005) program from the Academy for Advanced Interdisciplinary Studies of Henan University (Y21008L).

## Conflict of Interest

The authors declare that the research was conducted in the absence of any commercial or financial relationships that could be construed as a potential conflict of interest.

## Publisher’s Note

All claims expressed in this article are solely those of the authors and do not necessarily represent those of their affiliated organizations, or those of the publisher, the editors and the reviewers. Any product that may be evaluated in this article, or claim that may be made by its manufacturer, is not guaranteed or endorsed by the publisher.

## Abbreviations

GC, gastric cancer; GEO, Gene Expression Omnibus; TCGA, The Cancer Genome Atlas; SETD2, SET domain-containing protein 2; BC, breast cancer; OC, ovarian cancer; HCC, hepatocellular carcinoma; LUCA, lung carcinoma.
